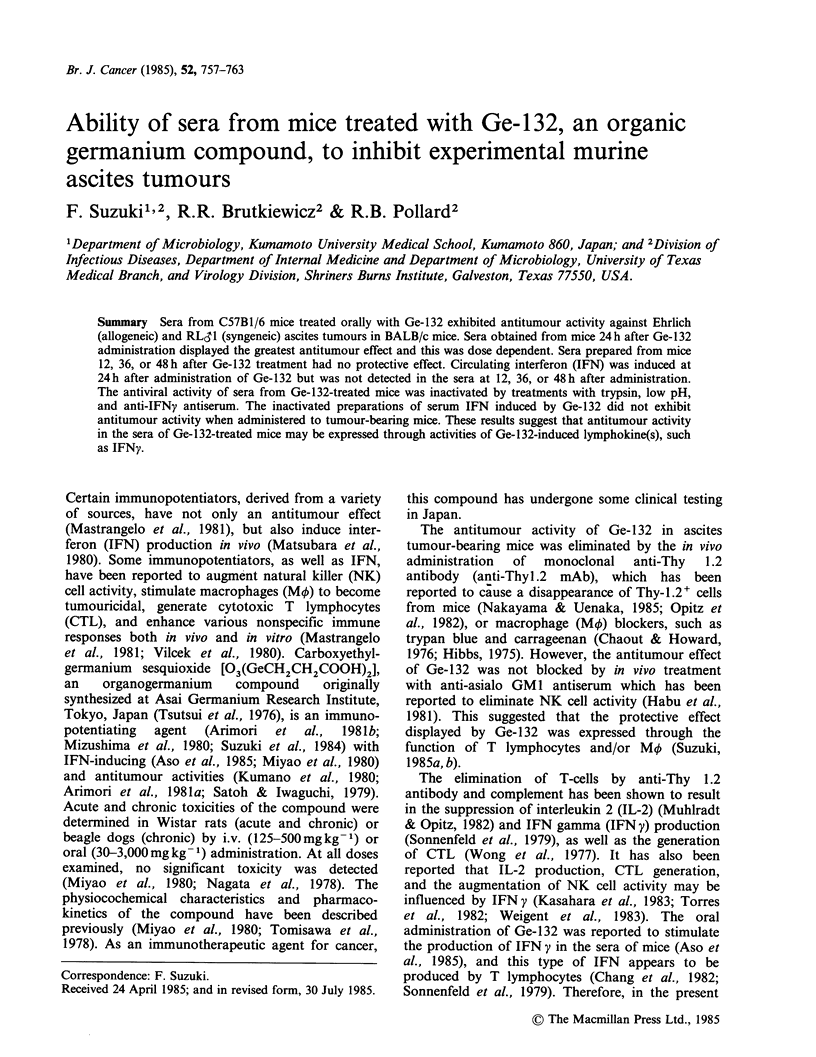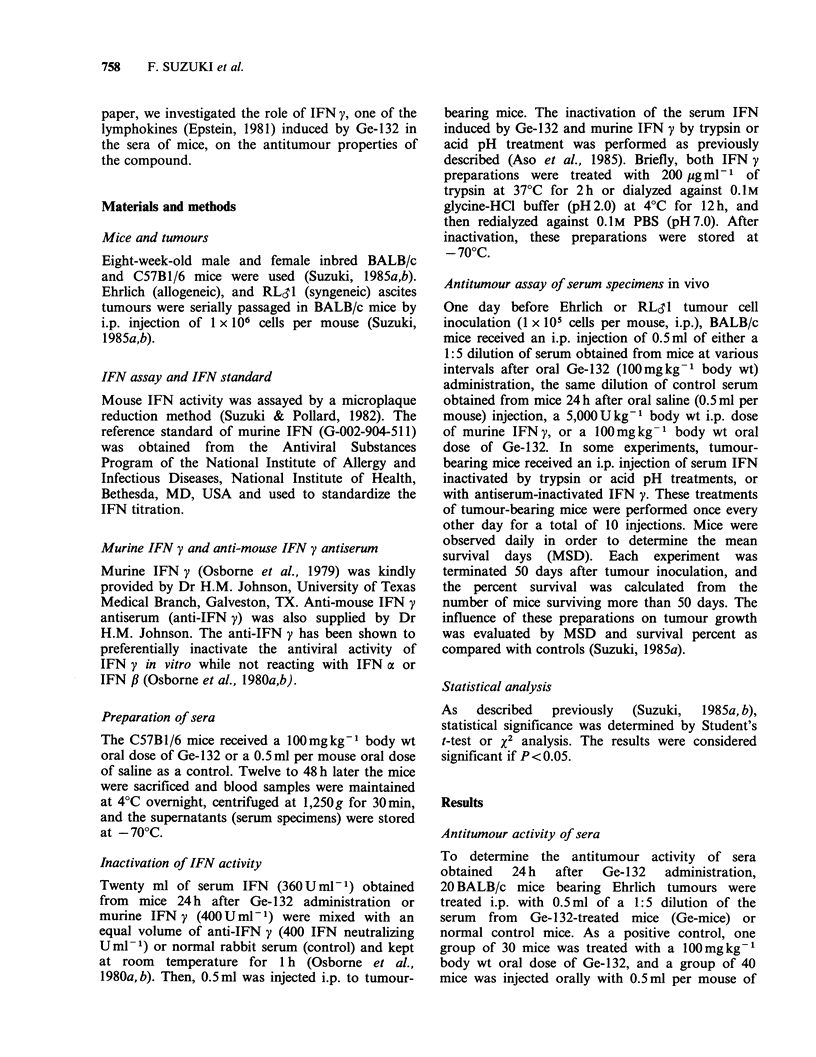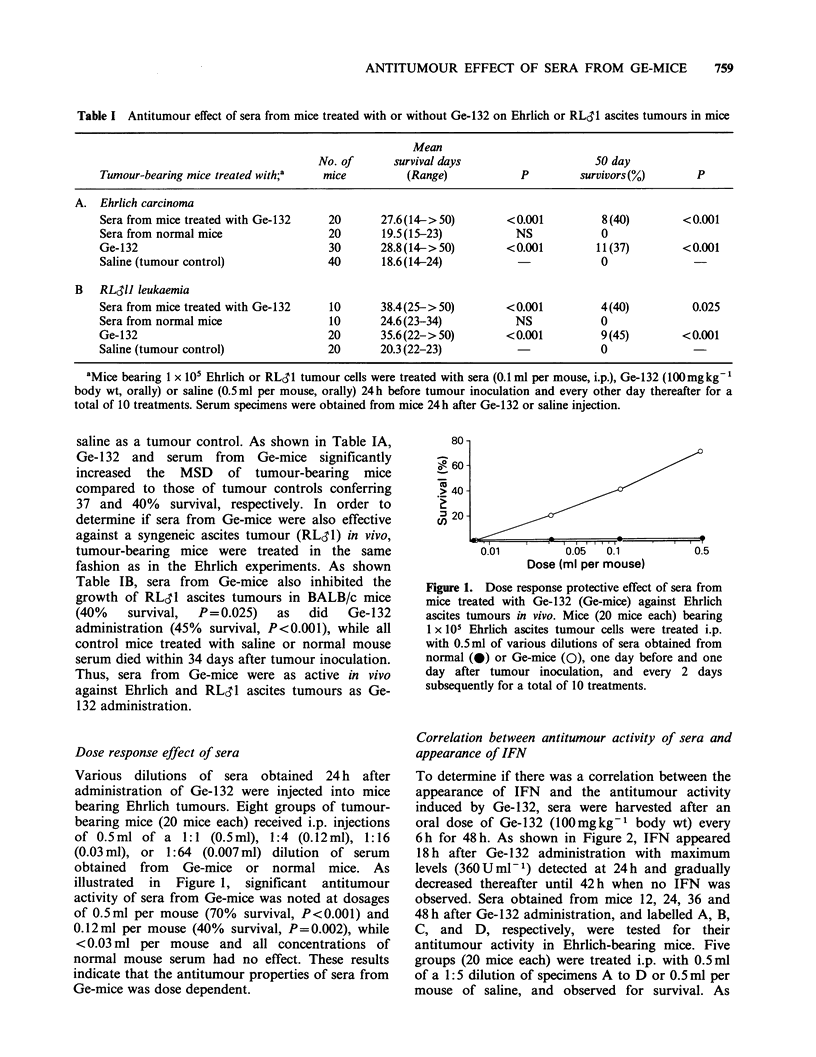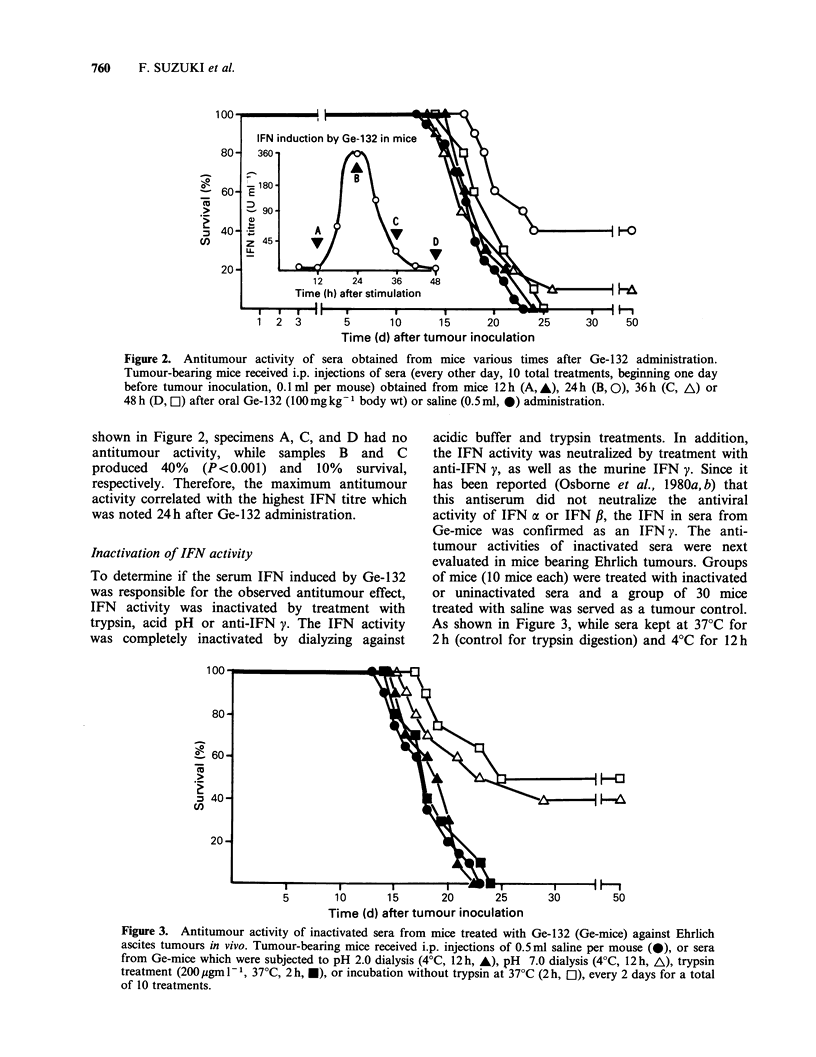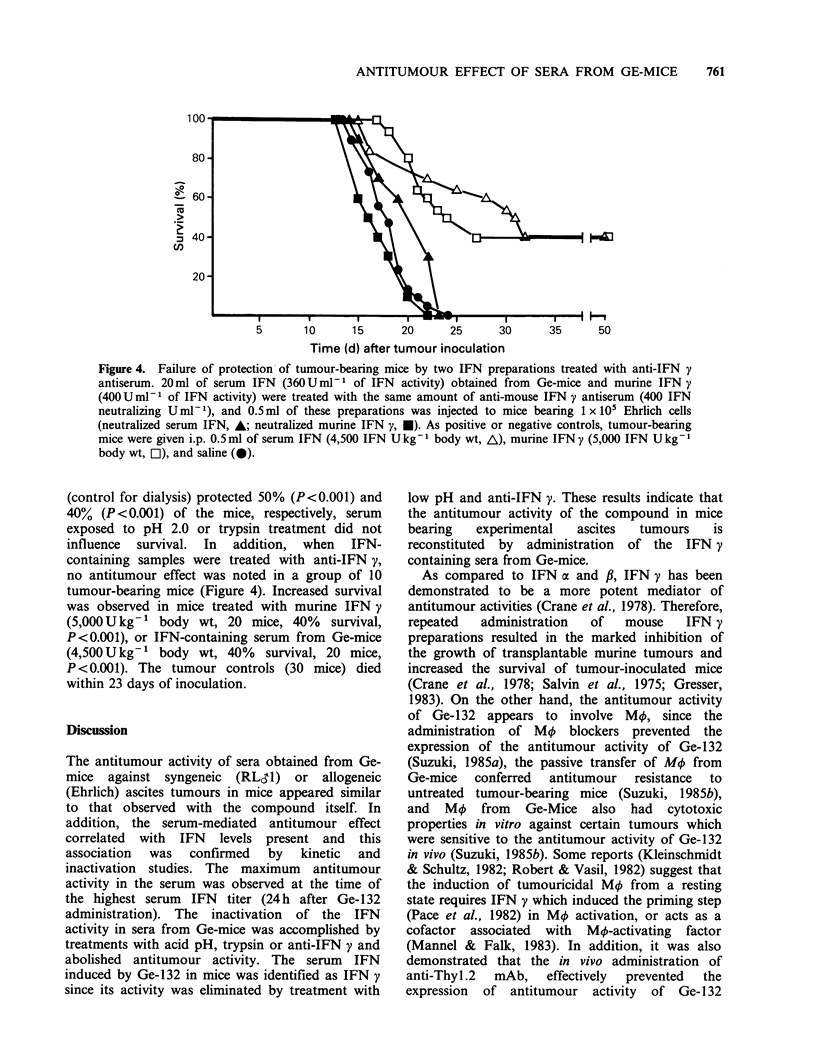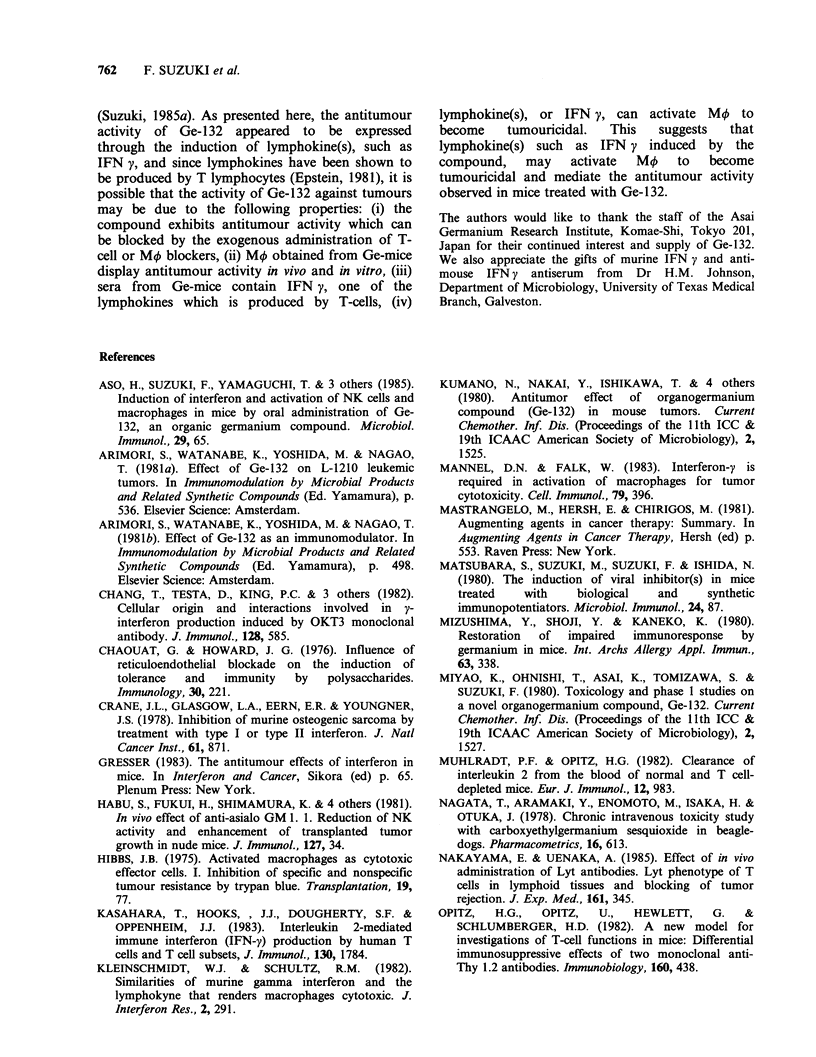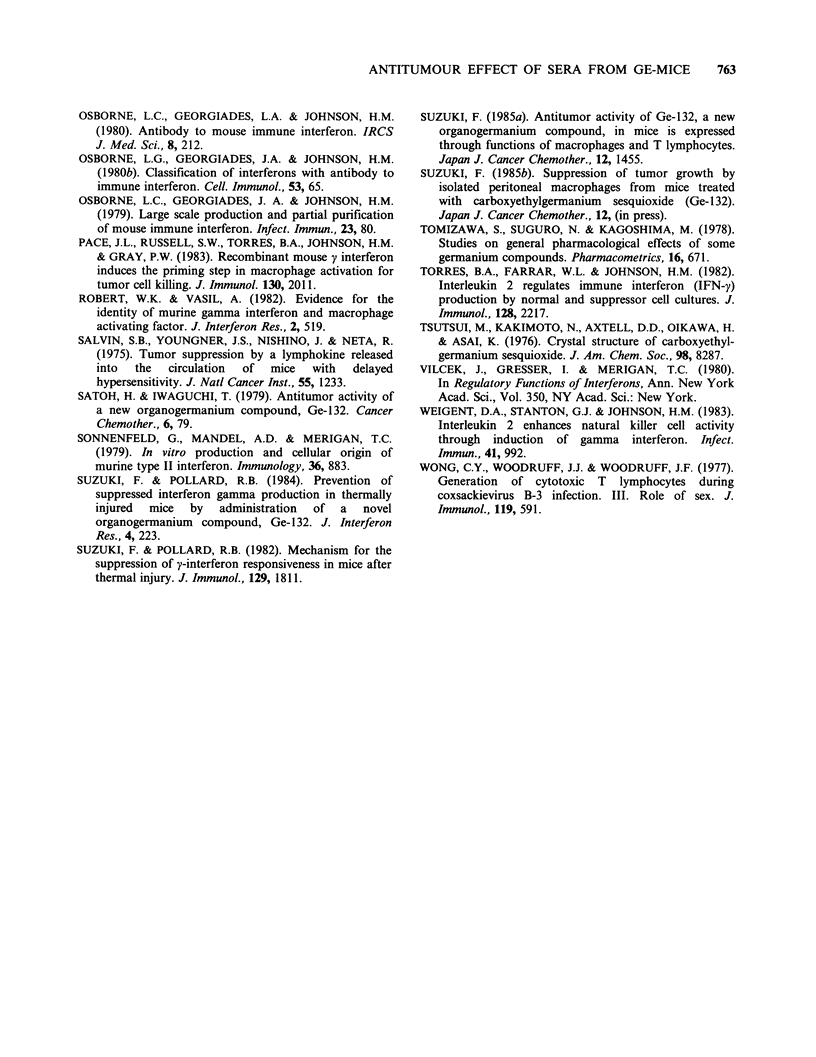# Ability of sera from mice treated with Ge-132, an organic germanium compound, to inhibit experimental murine ascites tumours.

**DOI:** 10.1038/bjc.1985.254

**Published:** 1985-11

**Authors:** F. Suzuki, R. R. Brutkiewicz, R. B. Pollard

## Abstract

Sera from C57Bl/6 mice treated orally with Ge-132 exhibited antitumour activity against Ehrlich (allogeneic) and RL male 1 (syngeneic) ascites tumours in BALB/c mice. Sera obtained from mice 24 h after Ge-132 administration displayed the greatest antitumour effect and this was dose dependent. Sera prepared from mice 12, 36, or 48 h after Ge-132 treatment had no protective effect. Circulating interferon (IFN) was induced at 24 h after administration of Ge-132 but was not detected in the sera at 12, 36, or 48 h after administration. The antiviral activity of sera from Ge-132-treated mice was inactivated by treatments with trypsin, low pH, and anti-IFN gamma antiserum. The inactivated preparations of serum IFN induced by Ge-132 did not exhibit antitumour activity when administered to tumour-bearing mice. These results suggest that antitumour activity in the sera of Ge-132-treated mice may be expressed through activities of Ge-132-induced lymphokine(s), such as IFN gamma.


*Br. J. Cancer* (1985), **52**, 757–763

### Ability of sera from mice treated with Ge-132, an organic germanium compound, to inhibit experimental murine ascites tumours

F. Suzuki[1,2], R.R. Brutkiewicz[2] & R.B. Pollard[2]

[1]*Department of Microbiology, Kumamoto University Medical School, Kumamoto 860, Japan; and* [2]*Division of Infectious Diseases, Department of Internal Medicine and Department of Microbiology, University of Texas Medical Branch, and Virology Division, Shriners Burns Institute, Galveston, Texas 77550, USA.*

**Summary** Sera from C57Bl/6 mice treated orally with Ge-132 exhibited antitumour activity against Ehrlich (allogeneic) and RL♂1 (syngeneic) ascites tumours in BALB/c mice. Sera obtained from mice 24 h after Ge-132 administration displayed the greatest antitumour effect and this was dose dependent. Sera prepared from mice 12, 36, or 48 h after Ge-132 treatment had no protective effect. Circulating interferon (IFN) was induced at 24 h after administration of Ge-132 but was not detected in the sera at 12, 36, or 48 h after administration. The antiviral activity of sera from Ge-132-treated mice was inactivated by treatments with trypsin, low pH, and anti-IFNγ antiserum. The inactivated preparations of serum IFN induced by Ge-132 did not exhibit antitumour activity when administered to tumour-bearing mice. These results suggest that antitumour activity in the sera of Ge-132-treated mice may be expressed through activities of Ge-132-induced lymphokine(s), such as IFNγ.

Certain immunopotentiators, derived from a variety of sources, have not only an antitumour effect (Mastrangelo *et al.*, 1981), but also induce interferon (IFN) production *in vivo* (Matsubara *et al.*, 1980). Some immunopotentiators, as well as IFN, have been reported to augment natural killer (NK) cell activity, stimulate macrophages (Mϕ) to become tumouricidal, generate cytotoxic T lymphocytes (CTL), and enhance various nonspecific immune responses both *in vivo* and *in vitro* (Mastrangelo *et al.*, 1981; Vilcek *et al.*, 1980). Carboxyethylgermanium sesquioxide $[O_3(GeCH_2CH_2COOH)_2]$, an organogermanium compound originally synthesized at Asai Germanium Research Institute, Tokyo, Japan (Tsutsui *et al.*, 1976), is an immunopotentiating agent (Arimori *et al.*, 1981*b*; Mizushima *et al.*, 1980; Suzuki *et al.*, 1984) with IFN-inducing (Aso *et al.*, 1985; Miyao *et al.*, 1980) and antitumour activities (Kumano *et al.*, 1980; Arimori *et al.*, 1981*a*; Satoh & Iwaguchi, 1979). Acute and chronic toxicities of the compound were determined in Wistar rats (acute and chronic) or beagle dogs (chronic) by i.v. (125–500 mg $kg^{-1}$) or oral (30–3,000 mg $kg^{-1}$) administration. At all doses examined, no significant toxicity was detected (Miyao *et al.*, 1980; Nagata *et al.*, 1978). The physiocochemical characteristics and pharmacokinetics of the compound have been described previously (Miyao *et al.*, 1980; Tomisawa *et al.*, 1978). As an immunotherapeutic agent for cancer, this compound has undergone some clinical testing in Japan.

The antitumour activity of Ge-132 in ascites tumour-bearing mice was eliminated by the *in vivo* administration of monoclonal anti-Thy 1.2 antibody (anti-Thy1.2 mAb), which has been reported to cause a disappearance of Thy-1.2$^+$ cells from mice (Nakayama & Uenaka, 1985; Opitz *et al.*, 1982), or macrophage (Mϕ) blockers, such as trypan blue and carrageenan (Chaout & Howard, 1976; Hibbs, 1975). However, the antitumour effect of Ge-132 was not blocked by *in vivo* treatment with anti-asialo GM1 antiserum which has been reported to eliminate NK cell activity (Habu *et al.*, 1981). This suggested that the protective effect displayed by Ge-132 was expressed through the function of T lymphocytes and/or Mϕ (Suzuki, 1985*a*, *b*).

The elimination of T-cells by anti-Thy 1.2 antibody and complement has been shown to result in the suppression of interleukin 2 (IL-2) (Muhlradt & Opitz, 1982) and IFN gamma (IFNγ) production (Sonnenfeld *et al.*, 1979), as well as the generation of CTL (Wong *et al.*, 1977). It has also been reported that IL-2 production, CTL generation, and the augmentation of NK cell activity may be influenced by IFNγ (Kasahara *et al.*, 1983; Torres *et al.*, 1982; Weigent *et al.*, 1983). The oral administration of Ge-132 was reported to stimulate the production of IFNγ in the sera of mice (Aso *et al.*, 1985), and this type of IFN appears to be produced by T lymphocytes (Chang *et al.*, 1982; Sonnenfeld *et al.*, 1979). Therefore, in the present

Correspondence: F. Suzuki.
Received 24 April 1985; and in revised form, 30 July 1985.

© The Macmillan Press Ltd., 1985

paper, we investigated the role of IFN $\gamma$, one of the lymphokines (Epstein, 1981) induced by Ge-132 in the sera of mice, on the antitumour properties of the compound.

### Materials and methods

### *Mice and tumours*

Eight-week-old male and female inbred BALB/c and C57B1/6 mice were used (Suzuki, 1985*a,b*). Ehrlich (allogeneic), and RL♂1 (syngeneic) ascites tumours were serially passaged in BALB/c mice by i.p. injection of $1 \times 10^6$ cells per mouse (Suzuki, 1985*a,b*).

### *IFN assay and IFN standard*

Mouse IFN activity was assayed by a microplaque reduction method (Suzuki & Pollard, 1982). The reference standard of murine IFN (G-002-904-511) was obtained from the Antiviral Substances Program of the National Institute of Allergy and Infectious Diseases, National Institute of Health, Bethesda, MD, USA and used to standardize the IFN titration.

### *Murine IFN $\gamma$ and anti-mouse IFN $\gamma$ antiserum*

Murine IFN $\gamma$ (Osborne *et al.*, 1979) was kindly provided by Dr H.M. Johnson, University of Texas Medical Branch, Galveston, TX. Anti-mouse IFN $\gamma$ antiserum (anti-IFN $\gamma$) was also supplied by Dr H.M. Johnson. The anti-IFN $\gamma$ has been shown to preferentially inactivate the antiviral activity of IFN $\gamma$ *in vitro* while not reacting with IFN $\alpha$ or IFN $\beta$ (Osborne *et al.*, 1980*a,b*).

### *Preparation of sera*

The C57B1/6 mice received a $100\,\mathrm{mg\,kg^{-1}}$ body wt oral dose of Ge-132 or a 0.5 ml per mouse oral dose of saline as a control. Twelve to 48 h later the mice were sacrificed and blood samples were maintained at 4°C overnight, centrifuged at 1,250 *g* for 30 min, and the supernatants (serum specimens) were stored at −70°C.

### *Inactivation of IFN activity*

Twenty ml of serum IFN ($360\,\mathrm{U\,ml^{-1}}$) obtained from mice 24 h after Ge-132 administration or murine IFN $\gamma$ ($400\,\mathrm{U\,ml^{-1}}$) were mixed with an equal volume of anti-IFN $\gamma$ (400 IFN neutralizing $\mathrm{U\,ml^{-1}}$) or normal rabbit serum (control) and kept at room temperature for 1 h (Osborne *et al.*, 1980*a, b*). Then, 0.5 ml was injected i.p. to tumour-bearing mice. The inactivation of the serum IFN induced by Ge-132 and murine IFN $\gamma$ by trypsin or acid pH treatment was performed as previously described (Aso *et al.*, 1985). Briefly, both IFN $\gamma$ preparations were treated with $200\,\mu\mathrm{g\,ml^{-1}}$ of trypsin at 37°C for 2 h or dialyzed against 0.1 M glycine-HCl buffer (pH 2.0) at 4°C for 12 h, and then redialyzed against 0.1 M PBS (pH 7.0). After inactivation, these preparations were stored at −70°C.

### *Antitumour assay of serum specimens* in vivo

One day before Ehrlich or RL♂1 tumour cell inoculation ($1 \times 10^5$ cells per mouse, i.p.), BALB/c mice received an i.p. injection of 0.5 ml of either a 1:5 dilution of serum obtained from mice at various intervals after oral Ge-132 ($100\,\mathrm{mg\,kg^{-1}}$ body wt) administration, the same dilution of control serum obtained from mice 24 h after oral saline (0.5 ml per mouse) injection, a $5{,}000\,\mathrm{U\,kg^{-1}}$ body wt i.p. dose of murine IFN $\gamma$, or a $100\,\mathrm{mg\,kg^{-1}}$ body wt oral dose of Ge-132. In some experiments, tumour-bearing mice received an i.p. injection of serum IFN inactivated by trypsin or acid pH treatments, or with antiserum-inactivated IFN $\gamma$. These treatments of tumour-bearing mice were performed once every other day for a total of 10 injections. Mice were observed daily in order to determine the mean survival days (MSD). Each experiment was terminated 50 days after tumour inoculation, and the percent survival was calculated from the number of mice surviving more than 50 days. The influence of these preparations on tumour growth was evaluated by MSD and survival percent as compared with controls (Suzuki, 1985*a*).

### *Statistical analysis*

As described previously (Suzuki, 1985*a, b*), statistical significance was determined by Student's *t*-test or $\chi^2$ analysis. The results were considered significant if $P < 0.05$.

### Results

### *Antitumour activity of sera*

To determine the antitumour activity of sera obtained 24 h after Ge-132 administration, 20 BALB/c mice bearing Ehrlich tumours were treated i.p. with 0.5 ml of a 1:5 dilution of the serum from Ge-132-treated mice (Ge-mice) or normal control mice. As a positive control, one group of 30 mice was treated with a $100\,\mathrm{mg\,kg^{-1}}$ body wt oral dose of Ge-132, and a group of 40 mice was injected orally with 0.5 ml per mouse of

**Table I** Antitumour effect of sera from mice treated with or without Ge-132 on Ehrlich or RL♂1 ascites tumours in mice

| *Tumour-bearing mice treated with;*[a] | *No. of mice* | *Mean survival days (Range)* | *P* | *50 day survivors (%)* | *P* |
|---|---|---|---|---|---|
| A. *Ehrlich carcinoma* | | | | | |
| Sera from mice treated with Ge-132 | 20 | 27.6 (14->50) | <0.001 | 8 (40) | <0.001 |
| Sera from normal mice | 20 | 19.5 (15–23) | NS | 0 | |
| Ge-132 | 30 | 28.8 (14->50) | <0.001 | 11 (37) | <0.001 |
| Saline (tumour control) | 40 | 18.6 (14–24) | — | 0 | — |
| B *RL♂1l leukaemia* | | | | | |
| Sera from mice treated with Ge-132 | 10 | 38.4 (25->50) | <0.001 | 4 (40) | 0.025 |
| Sera from normal mice | 10 | 24.6 (23–34) | NS | 0 | |
| Ge-132 | 20 | 35.6 (22->50) | <0.001 | 9 (45) | <0.001 |
| Saline (tumour control) | 20 | 20.3 (22–23) | — | 0 | — |

[a]Mice bearing $1 \times 10^5$ Ehrlich or RL♂1 tumour cells were treated with sera (0.1 ml per mouse, i.p.), Ge-132 (100 mg kg$^{-1}$ body wt, orally) or saline (0.5 ml per mouse, orally) 24 h before tumour inoculation and every other day thereafter for a total of 10 treatments. Serum specimens were obtained from mice 24 h after Ge-132 or saline injection.

saline as a tumour control. As shown in Table IA, Ge-132 and serum from Ge-mice significantly increased the MSD of tumour-bearing mice compared to those of tumour controls conferring 37 and 40% survival, respectively. In order to determine if sera from Ge-mice were also effective against a syngeneic ascites tumour (RL♂1) *in vivo*, tumour-bearing mice were treated in the same fashion as in the Ehrlich experiments. As shown Table IB, sera from Ge-mice also inhibited the growth of RL♂1 ascites tumours in BALB/c mice (40% survival, $P=0.025$) as did Ge-132 administration (45% survival, $P<0.001$), while all control mice treated with saline or normal mouse serum died within 34 days after tumour inoculation. Thus, sera from Ge-mice were as active *in vivo* against Ehrlich and RL♂1 ascites tumours as Ge-132 administration.

**Figure 1.** Dose response protective effect of sera from mice treated with Ge-132 (Ge-mice) against Ehrlich ascites tumours *in vivo*. Mice (20 mice each) bearing $1 \times 10^5$ Ehrlich ascites tumour cells were treated i.p. with 0.5 ml of various dilutions of sera obtained from normal (●) or Ge-mice (○), one day before and one day after tumour inoculation, and every 2 days subsequently for a total of 10 treatments.

### *Dose response effect of sera*

Various dilutions of sera obtained 24 h after administration of Ge-132 were injected into mice bearing Ehrlich tumours. Eight groups of tumour-bearing mice (20 mice each) received i.p. injections of 0.5 ml of a 1:1 (0.5 ml), 1:4 (0.12 ml), 1:16 (0.03 ml), or 1:64 (0.007 ml) dilution of serum obtained from Ge-mice or normal mice. As illustrated in Figure 1, significant antitumour activity of sera from Ge-mice was noted at dosages of 0.5 ml per mouse (70% survival, $P<0.001$) and 0.12 ml per mouse (40% survival, $P=0.002$), while <0.03 ml per mouse and all concentrations of normal mouse serum had no effect. These results indicate that the antitumour properties of sera from Ge-mice was dose dependent.

### *Correlation between antitumour activity of sera and appearance of IFN*

To determine if there was a correlation between the appearance of IFN and the antitumour activity induced by Ge-132, sera were harvested after an oral dose of Ge-132 (100 mg kg$^{-1}$ body wt) every 6 h for 48 h. As shown in Figure 2, IFN appeared 18 h after Ge-132 administration with maximum levels (360 U ml$^{-1}$) detected at 24 h and gradually decreased thereafter until 42 h when no IFN was observed. Sera obtained from mice 12, 24, 36 and 48 h after Ge-132 administration, and labelled A, B, C, and D, respectively, were tested for their antitumour activity in Ehrlich-bearing mice. Five groups (20 mice each) were treated i.p. with 0.5 ml of a 1:5 dilution of specimens A to D or 0.5 ml per mouse of saline, and observed for survival. As

**Figure 2.** Antitumour activity of sera obtained from mice various times after Ge-132 administration. Tumour-bearing mice received i.p. injections of sera (every other day, 10 total treatments, beginning one day before tumour inoculation, 0.1 ml per mouse) obtained from mice 12 h (A, ▲), 24 h (B, ○), 36 h (C, △) or 48 h (D, □) after oral Ge-132 (100 mg kg$^{-1}$ body wt) or saline (0.5 ml, ●) administration.

shown in Figure 2, specimens A, C, and D had no antitumour activity, while samples B and C produced 40% ($P<0.001$) and 10% survival, respectively. Therefore, the maximum antitumour activity correlated with the highest IFN titre which was noted 24 h after Ge-132 administration.

### *Inactivation of IFN activity*

To determine if the serum IFN induced by Ge-132 was responsible for the observed antitumour effect, IFN activity was inactivated by treatment with trypsin, acid pH or anti-IFN $\gamma$. The IFN activity was completely inactivated by dialyzing against acidic buffer and trypsin treatments. In addition, the IFN activity was neutralized by treatment with anti-IFN $\gamma$, as well as the murine IFN $\gamma$. Since it has been reported (Osborne *et al.*, 1980*a*, *b*) that this antiserum did not neutralize the antiviral activity of IFN $\alpha$ or IFN $\beta$, the IFN in sera from Ge-mice was confirmed as an IFN $\gamma$. The antitumour activities of inactivated sera were next evaluated in mice bearing Ehrlich tumours. Groups of mice (10 mice each) were treated with inactivated or uninactivated sera and a group of 30 mice treated with saline was served as a tumour control. As shown in Figure 3, while sera kept at 37°C for 2 h (control for trypsin digestion) and 4°C for 12 h

**Figure 3.** Antitumour activity of inactivated sera from mice treated with Ge-132 (Ge-mice) against Ehrlich ascites tumours *in vivo*. Tumour-bearing mice received i.p. injections of 0.5 ml saline per mouse (●), or sera from Ge-mice which were subjected to pH 2.0 dialysis (4°C, 12 h, ▲), pH 7.0 dialysis (4°C, 12 h, △), trypsin treatment (200 μg ml$^{-1}$, 37°C, 2 h, ■), or incubation without trypsin at 37°C (2 h, □), every 2 days for a total of 10 treatments.

**Figure 4.** Failure of protection of tumour-bearing mice by two IFN preparations treated with anti-IFN $\gamma$ antiserum. 20 ml of serum IFN (360 U ml$^{-1}$ of IFN activity) obtained from Ge-mice and murine IFN $\gamma$ (400 U ml$^{-1}$ of IFN activity) were treated with the same amount of anti-mouse IFN $\gamma$ antiserum (400 IFN neutralizing U ml$^{-1}$), and 0.5 ml of these preparations was injected to mice bearing $1 \times 10^5$ Ehrlich cells (neutralized serum IFN, ▲; neutralized murine IFN $\gamma$, ■). As positive or negative controls, tumour-bearing mice were given i.p. 0.5 ml of serum IFN (4,500 IFN U kg$^{-1}$ body wt, △), murine IFN $\gamma$ (5,000 IFN U kg$^{-1}$ body wt, □), and saline (●).

(control for dialysis) protected 50% ($P<0.001$) and 40% ($P<0.001$) of the mice, respectively, serum exposed to pH 2.0 or trypsin treatment did not influence survival. In addition, when IFN-containing samples were treated with anti-IFN $\gamma$, no antitumour effect was noted in a group of 10 tumour-bearing mice (Figure 4). Increased survival was observed in mice treated with murine IFN $\gamma$ (5,000 U kg$^{-1}$ body wt, 20 mice, 40% survival, $P<0.001$), or IFN-containing serum from Ge-mice (4,500 U kg$^{-1}$ body wt, 40% survival, 20 mice, $P<0.001$). The tumour controls (30 mice) died within 23 days of inoculation.

### Discussion

The antitumour activity of sera obtained from Ge-mice against syngeneic (RL♂1) or allogeneic (Ehrlich) ascites tumours in mice appeared similar to that observed with the compound itself. In addition, the serum-mediated antitumour effect correlated with IFN levels present and this association was confirmed by kinetic and inactivation studies. The maximum antitumour activity in the serum was observed at the time of the highest serum IFN titer (24 h after Ge-132 administration). The inactivation of the IFN activity in sera from Ge-mice was accomplished by treatments with acid pH, trypsin or anti-IFN $\gamma$ and abolished antitumour activity. The serum IFN induced by Ge-132 in mice was identified as IFN $\gamma$ since its activity was eliminated by treatment with low pH and anti-IFN $\gamma$. These results indicate that the antitumour activity of the compound in mice bearing experimental ascites tumours is reconstituted by administration of the IFN $\gamma$ containing sera from Ge-mice.

As compared to IFN $\alpha$ and $\beta$, IFN $\gamma$ has been demonstrated to be a more potent mediator of antitumour activities (Crane *et al.*, 1978). Therefore, repeated administration of mouse IFN $\gamma$ preparations resulted in the marked inhibition of the growth of transplantable murine tumours and increased the survival of tumour-inoculated mice (Crane *et al.*, 1978; Salvin *et al.*, 1975; Gresser, 1983). On the other hand, the antitumour activity of Ge-132 appears to involve M$\phi$, since the administration of M$\phi$ blockers prevented the expression of the antitumour activity of Ge-132 (Suzuki, 1985*a*), the passive transfer of *M$\phi$* from Ge-mice conferred antitumour resistance to untreated tumour-bearing mice (Suzuki, 1985*b*), and M$\phi$ from Ge-Mice also had cytotoxic properties *in vitro* against certain tumours which were sensitive to the antitumour activity of Ge-132 *in vivo* (Suzuki, 1985*b*). Some reports (Kleinschmidt & Schultz, 1982; Robert & Vasil, 1982) suggest that the induction of tumouricidal M$\phi$ from a resting state requires IFN $\gamma$ which induced the priming step (Pace *et al.*, 1982) in M$\phi$ activation, or acts as a cofactor associated with M$\phi$-activating factor (Mannel & Falk, 1983). In addition, it was also demonstrated that the *in vivo* administration of anti-Thy1.2 mAb, effectively prevented the expression of antitumour activity of Ge-132

(Suzuki, 1985a). As presented here, the antitumour activity of Ge-132 appeared to be expressed through the induction of lymphokine(s), such as IFN γ, and since lymphokines have been shown to be produced by T lymphocytes (Epstein, 1981), it is possible that the activity of Ge-132 against tumours may be due to the following properties: (i) the compound exhibits antitumour activity which can be blocked by the exogenous administration of T-cell or Mϕ blockers, (ii) Mϕ obtained from Ge-mice display antitumour activity *in vivo* and *in vitro*, (iii) sera from Ge-mice contain IFN γ, one of the lymphokines which is produced by T-cells, (iv) lymphokine(s), or IFN γ, can activate Mϕ to become tumouricidal. This suggests that lymphokine(s) such as IFN γ induced by the compound, may activate Mϕ to become tumouricidal and mediate the antitumour activity observed in mice treated with Ge-132.

The authors would like to thank the staff of the Asai Germanium Research Institute, Komae-Shi, Tokyo 201, Japan for their continued interest and supply of Ge-132. We also appreciate the gifts of murine IFN γ and anti-mouse IFN γ antiserum from Dr H.M. Johnson, Department of Microbiology, University of Texas Medical Branch, Galveston.

### References

ASO, H., SUZUKI, F., YAMAGUCHI, T. & 3 others (1985). Induction of interferon and activation of NK cells and macrophages in mice by oral administration of Ge-132, an organic germanium compound. *Microbiol. Immunol.*, **29**, 65.

ARIMORI, S., WATANABE, K., YOSHIDA, M. & NAGAO, T. (1981*a*). Effect of Ge-132 on L-1210 leukemic tumors. In *Immunomodulation by Microbial Products and Related Synthetic Compounds* (Ed. Yamamura), p. 536. Elsevier Science: Amsterdam.

ARIMORI, S., WATANABE, K., YOSHIDA, M. & NAGAO, T. (1981*b*). Effect of Ge-132 as an immunomodulator. In *Immunomodulation by Microbial Products and Related Synthetic Compounds* (Ed. Yamamura), p. 498. Elsevier Science: Amsterdam.

CHANG, T., TESTA, D., KING, P.C. & 3 others (1982). Cellular origin and interactions involved in γ-interferon production induced by OKT3 monoclonal antibody. *J. Immunol.*, **128**, 585.

CHAOUAT, G. & HOWARD, J. G. (1976). Influence of reticuloendothelial blockade on the induction of tolerance and immunity by polysaccharides. *Immunology*, **30**, 221.

CRANE, J.L., GLASGOW, L.A., EERN, E.R. & YOUNGNER, J.S. (1978). Inhibition of murine osteogenic sarcoma by treatment with type I or type II interferon. *J. Natl Cancer Inst.*, **61**, 871.

GRESSER (1983). The antitumour effects of interferon in mice. In *Interferon and Cancer*, Sikora (ed) p. 65. Plenum Press: New York.

HABU, S., FUKUI, H., SHIMAMURA, K. & 4 others (1981). *In vivo* effect of anti-asialo GM 1. 1. Reduction of NK activity and enhancement of transplanted tumor growth in nude mice. *J. Immunol.*, **127**, 34.

HIBBS, J.B. (1975). Activated macrophages as cytotoxic effector cells. I. Inhibition of specific and nonspecific tumour resistance by trypan blue. *Transplantation*, **19**, 77.

KASAHARA, T., HOOKS, , J.J., DOUGHERTY, S.F. & OPPENHEIM, J.J. (1983). Interleukin 2-mediated immune interferon (IFN-γ) production by human T cells and T cell subsets, *J. Immunol.*, **130**, 1784.

KLEINSCHMIDT, W.J. & SCHULTZ, R.M. (1982). Similarities of murine gamma interferon and the lymphokyne that renders macrophages cytotoxic. *J. Interferon Res.*, **2**, 291.

KUMANO, N., NAKAI, Y., ISHIKAWA, T. & 4 others (1980). Antitumor effect of organogermanium compound (Ge-132) in mouse tumors. *Current Chemother. Inf. Dis.* (Proceedings of the 11th ICC & 19th ICAAC American Society of Microbiology), **2**, 1525.

MANNEL, D.N. & FALK, W. (1983). Interferon-γ is required in activation of macrophages for tumor cytotoxicity. *Cell. Immunol.*, **79**, 396.

MASTRANGELO, M., HERSH, E. & CHIRIGOS, M. (1981). Augmenting agents in cancer therapy: Summary. In *Augmenting Agents in Cancer Therapy*, Hersh (ed) p. 553. Raven Press: New York.

MATSUBARA, S., SUZUKI, M., SUZUKI, F. & ISHIDA, N. (1980). The induction of viral inhibitor(s) in mice treated with biological and synthetic immunopotentiators. *Microbiol. Immunol.*, **24**, 87.

MIZUSHIMA, Y., SHOJI, Y. & KANEKO, K. (1980). Restoration of impaired immunoresponse by germanium in mice. *Int. Archs Allergy Appl. Immun.*, **63**, 338.

MIYAO, K., OHNISHI, T., ASAI, K., TOMIZAWA, S. & SUZUKI, F. (1980). Toxicology and phase 1 studies on a novel organogermanium compound, Ge-132. *Current Chemother. Inf. Dis.* (Proceedings of the 11th ICC & 19th ICAAC American Society of Microbiology), **2**, 1527.

MUHLRADT, P.F. & OPITZ, H.G. (1982). Clearance of interleukin 2 from the blood of normal and T cell-depleted mice. *Eur. J. Immunol.*, **12**, 983.

NAGATA, T., ARAMAKI, Y., ENOMOTO, M., ISAKA, H. & OTUKA, J. (1978). Chronic intravenous toxicity study with carboxyethylgermanium sesquioxide in beagle-dogs. *Pharmacometrics*, **16**, 613.

NAKAYAMA, E. & UENAKA, A. (1985). Effect of *in vivo* administration of Lyt antibodies. Lyt phenotype of T cells in lymphoid tissues and blocking of tumor rejection. *J. Exp. Med.*, **161**, 345.

OPITZ, H.G., OPITZ, U., HEWLETT, G. & SCHLUMBERGER, H.D. (1982). A new model for investigations of T-cell functions in mice: Differential immunosuppressive effects of two monoclonal anti-Thy 1.2 antibodies. *Immunobiology*, **160**, 438.

OSBORNE, L.C., GEORGIADES, L.A. & JOHNSON, H.M. (1980). Antibody to mouse immune interferon. *IRCS J. Med. Sci.*, **8**, 212.

OSBORNE, L.G., GEORGIADES, J.A. & JOHNSON, H.M. (1980*b*). Classification of interferons with antibody to immune interferon. *Cell. Immunol.*, **53**, 65.

OSBORNE, L.C., GEORGIADES, J. A. & JOHNSON, H.M. (1979). Large scale production and partial purification of mouse immune interferon. *Infect. Immun.*, **23**, 80.

PACE, J.L., RUSSELL, S.W., TORRES, B.A., JOHNSON, H.M. & GRAY, P.W. (1983). Recombinant mouse $\gamma$ interferon induces the priming step in macrophage activation for tumor cell killing. *J. Immunol.* **130**, 2011.

ROBERT, W.K. & VASIL, A. (1982). Evidence for the identity of murine gamma interferon and macrophage activating factor. *J. Interferon Res.*, **2**, 519.

SALVIN, S.B., YOUNGNER, J.S., NISHINO, J. & NETA, R. (1975). Tumor suppression by a lymphokine released into the circulation of mice with delayed hypersensitivity. *J. Natl Cancer Inst.*, **55**, 1233.

SATOH, H. & IWAGUCHI, T. (1979). Antitumor activity of a new organogermanium compound, Ge-132. *Cancer Chemother.*, **6**, 79.

SONNENFELD, G., MANDEL, A.D. & MERIGAN, T.C. (1979). *In vitro* production and cellular origin of murine type II interferon. *Immunology*, **36**, 883.

SUZUKI, F. & POLLARD, R.B. (1984). Prevention of suppressed interferon gamma production in thermally injured mice by administration of a novel organogermanium compound, Ge-132. *J. Interferon Res.*, **4**, 223.

SUZUKI, F. & POLLARD, R.B. (1982). Mechanism for the suppression of $\gamma$-interferon responsiveness in mice after thermal injury. *J. Immunol.*, **129**, 1811.

SUZUKI, F. (1985*a*). Antitumor activity of Ge-132, a new organogermanium compound, in mice is expressed through functions of macrophages and T lymphocytes. *Japan J. Cancer Chemother.*, **12**, 1455.

SUZUKI, F. (1985*b*). Suppression of tumor growth by isolated peritoneal macrophages from mice treated with carboxyethylgermanium sesquioxide (Ge-132). *Japan J. Cancer Chemother.*, **12**, (in press).

TOMIZAWA, S., SUGURO, N. & KAGOSHIMA, M. (1978). Studies on general pharmacological effects of some germanium compounds. *Pharmacometrics*, **16**, 671.

TORRES, B.A., FARRAR, W.L. & JOHNSON, H.M. (1982). Interleukin 2 regulates immune interferon (IFN-$\gamma$) production by normal and suppressor cell cultures. *J. Immunol.*, **128**, 2217.

TSUTSUI, M., KAKIMOTO, N., AXTELL, D.D., OIKAWA, H. & ASAI, K. (1976). Crystal structure of carboxyethylgermanium sesquioxide. *J. Am. Chem. Soc.*, **98**, 8287.

VILCEK, J., GRESSER, I. & MERIGAN, T.C. (1980). In *Regulatory Functions of Interferons*, Ann. New York Acad. Sci., Vol. 350, NY Acad. Sci.: New York.

WEIGENT, D.A., STANTON, G.J. & JOHNSON, H.M. (1983). Interleukin 2 enhances natural killer cell activity through induction of gamma interferon. *Infect. Immun.*, **41**, 992.

WONG, C.Y., WOODRUFF, J.J. & WOODRUFF, J.F. (1977). Generation of cytotoxic T lymphocytes during coxsackievirus B-3 infection. III. Role of sex. *J. Immunol.*, **119**, 591.